# The Potential Connection Between Mechanical Stress and Heart Failure with Preserved Ejection Fraction: Mechanistic Insights and Therapeutic Potential

**DOI:** 10.7150/ijms.118184

**Published:** 2026-01-01

**Authors:** Hong Yang, Hong Wang, Luyun Wang, Jiangang Jiang

**Affiliations:** 1 Division of Cardiology, Department of Internal Medicine, Tongji Hospital, Tongji Medical College, Huazhong University of Science and Technology, Wuhan 430030, PR China.; 2 Hubei Key Laboratory of Genetics and Molecular Mechanism of Cardiologic Disorders, Tongji Hospital, Tongji Medical College, Huazhong University of Science and Technology, Wuhan, 430000, P. R. China.

**Keywords:** mechanical stress, mechanosensation, cardiac remodeling, cardiac fibrosis, inflammation, endothelial dysfunction, mitochondrial, calcium imbalance, heart failure with preserved ejection fraction

## Abstract

Heart failure with preserved ejection fraction (HFpEF) represents a complex clinical syndrome characterized by limited therapeutic options, which are largely due to its intricate pathophysiology. The role of mechanical stress is pivotal in maintaining cardiovascular homeostasis; conversely, its dysregulation may precipitate the progression of cardiovascular diseases. In HFpEF, both macroscopic structural alterations and intricate molecular processes might be influenced by mechanical stress. This review examines the potential associations between mechanical stress and HFpEF, exploring the pathophysiological underpinnings to the effects of mechanotransduction effectors on cardiac remodeling and the progression of heart failure, providing novel insights into the pathological mechanisms of HFpEF. Therapeutic strategies targeting these mechanotransduction effectors have shown promise in mitigating pathological cardiac remodeling in models of metabolic-associated heart failure, underscoring their potential as innovative treatments for HFpEF. Considering the clinical heterogeneity of HFpEF, it is imperative to pursue phenotype-specific personalized treatments to optimize therapeutic efficacy.

## 1. Introduction

Heart failure is a complex clinical entity characterized by significantly increased morbidity and mortality, posing a substantial challenge in contemporary medical care^1^. It is categorized into three distinct subtypes based on left ventricular ejection fraction (LVEF): heart failure with preserved ejection fraction (HFpEF), heart failure with mid-range ejection fraction (HFmrEF), and heart failure with reduced ejection fraction (HFrEF)^2^. Traditionally, HFrEF has been the primary focus of research due to its well-defined pathophysiological mechanisms and the availability of effective therapeutic interventions. However, in recent years, the prevalence of HFpEF has surged, now equaling that of HFrEF, and has become a critical area within the field of heart failure that requires urgent attention^3^. Despite the similar prognosis for patients with HFpEF and HFrEF, effective treatment options for HFpEF remain scarce. This therapeutic gap primarily stems from the variability in pathophysiological mechanisms observed across the clinical spectrum of HFpEF, where distinct pathological subtypes result in different responses to treatment among patient populations^4^. Central to the pathophysiology of HFpEF is diastolic impairment, mediated by increased ventricular stiffness due to maladaptive responses to biomechanical stress, including pressure overload, aberrant myocardial strain patterns, and pathological extracellular matrix (ECM) remodeling^5^. Therefore, improving cardiac performance in HFpEF can be achieved by enhancing diastolic filling through the reduction of myocardial stiffness, which increases diastolic volume and stroke volume via the Frank-Starling mechanism^6^.

Cardiomyocytes are situated within a specialized mechanobiological niche, incessantly exposed to dynamic mechanical stimuli throughout the cardiac cycle. This mechanical milieu comprises chronic physiological hemodynamic forces, variations in chamber pressure, alterations in tissue morphology, and modifications in contractile stretch^7^, ^8^. These mechanical stimuli are transmitted through the integrated myofibrillar ECM network, facilitating the generation of ventricular pressure and the maintenance of cardiac output^9^. Mechanical signals and forces are conveyed into cells via mechanosensitive channels, thus regulating cardiac pathophysiological functions. Under physiological conditions, mechanical stress is essential for sustaining the normal physiological processes in cardiomyocytes, including proliferation, differentiation, and maturation. The heart adjusts to dynamic mechanical environments through adaptive cardiac remodeling, thereby preserving stable cardiac function^10^. However, prolonged mechanical overload triggers maladaptive signaling cascades and pathological remodeling, leading to altered myocardial architecture and biomechanical properties. Such alterations disrupt the force equilibrium across myocardial regions and extracellular components^11^. In the context of common comorbidities associated with HFpEF, obesity promotes plasma volume expansion, biventricular structural remodeling, increased accumulation of epicardial adipose tissue, and enhanced pericardial constraint^12^. These changes lead to alterations in ventricular tensile stress and wall stress, whereby pericardial restraint and ventricular remodeling reduce ventricular compliance, culminating in mechanical injury^13^. Diabetes mellitus induces microvascular dysfunction, alters cardiac shear stress patterns, and exacerbates vascular stiffening, collectively contributing to adverse ventricular remodeling^12^, ^14^. Hypertension increases vascular wall pressure and ventricular afterload, while also modifying myocardial tensile stress and vascular wall shear stress^15^. Elevated left ventricular filling pressures augment wall stress, fostering collagen deposition and diastolic dysfunction^16^. Zile *et al.* demonstrated that the transition from hypertension to HFpEF involves pathological modifications of both the collagen matrix and titin^5^. Titin, a colossal sarcomeric protein that regulates passive tension and possesses mechanical activity^17^, exhibits modifiable elastic properties through phosphorylation and isoform composition. Dysregulation of these properties increases resting stiffness, further impairing ventricular compliance^5^. Consequently, mechanical stress is pivotal in the pathogenesis and progression of HFpEF, contributing to cardiac remodeling and the development of heart failure through both extracellular force response and intracellular mechanotransduction pathways^9^. Regrettably, the prevailing comprehension of HFpEF often underestimates the critical role of mechanical stress in its development.

This review explores the potential association and role of mechanical stress in the pathogenesis of HFpEF, offering a novel perspective for elucidating the underlying pathophysiological mechanisms of this condition.

## 2. Classification of Mechanical Stress and Physiological Effects on the Heart

Mechanical stress encompasses a variety of biomechanical forces, including tension, stretch, and shear. These forces collectively regulate cardiac development, structural adaptation, and functional homeostasis^8^, ^18^.

### 2.1. Classification of mechanical stress in the heart

The heart is perpetually subjected to several principal mechanical forces. These include cyclic stretch, which refers to the phasic tensile and compressive stresses that occur during systolic contraction and diastolic relaxation^19^; shear stress, a tangential force exerted parallel to the surfaces of cardiomyocytes, primarily arising from the frictional force of blood flow against the vessel wall^20^; and hydrostatic pressure, a perpendicular force induced by the gravitational influence of blood within the cardiac chambers^21^. Additionally, the ECM consists of a complex fibrous network that surrounds cardiomyocytes. This matrix is primarily composed of collagen, fibronectin, elastin, laminin, proteoglycans, glycoproteins, and glycosaminoglycans. While fibrillar proteins are characterized by high tensile strength but low elasticity, elastic fibers display high elasticity with correspondingly low tensile strength. Consequently, the composition of the ECM significantly influences the mechanical stresses experienced by cardiac tissue. Under physiological conditions, ECM mitigates the mechanical stress generated by myocardial contraction through its elastic and tensile properties, maintaining the structural stability of myocardial cells^22^. These biomechanical factors are critical in governing cardiovascular embryogenesis, maintaining adult cardiac functional integrity, homeostatic regulation, and modulation of disease progression^18^, ^23^.

### 2.2 Adaptive modulation of heart structure and function by mechanical stress

During the initial stages of cardiogenesis, a specific type of cardiogenic mesodermal mesenchymal cell migrates to the ventral midline, contributing to the formation of the primitive heart tube^24^. This structure comprises an outer layer of myocardial cells and an inner layer of endocardial cells. The ECM accumulates between the endocardium and myocardium, providing crucial structural support and biomechanical stimuli to the developing myocardium through intracardiac pressure^25^. Heart tube looping involves two mechanistically distinct phases: c-looping, characterized by ventral bending and rightward rotation primarily driven by extrinsic mechanical forces, and s-looping, during which the primitive ventricle shifts caudally, and the distance between the outflow tract (OFT) and atrium decreases^25^, ^26^. Following the completion of s-looping, the primitive heart tube gradually differentiates into the systemic and pulmonary circuits. The orchestration of heart trabeculae and chamber formation is facilitated under hemodynamic forces^27^.

During cardiac development, the endothelial-to-mesenchymal transition (EndoMT) of endothelial cells is crucial for valvulogenesis^28^. Recent studies have revealed that the activation of klf2a via transient receptor potential (TRP) channels, and adenosine triphosphate (ATP)-mediated calcium (Ca^2+^) oscillations initiates nuclear factor of activated T-cells, cytoplasmic 1 (Nfatc1) signaling in response to localized high shear stress. This mechanotransduction cascade effectively converts mechanical stimuli into biochemical signals within endocardial cells, thus facilitating EndoMT and shaping of cardiac valves^29^. As the heart has evolved from a single-chambered to a multi-chambered structure, cardiac valves have developed to ensure unidirectional blood flow throughout the cardiac cycle. Concurrently, ventricular cardiomyocytes have organized into fibrous structures, creating complex laminar flow patterns within the cardiac walls, which impart essential mechanical properties. In the mature heart, mechanical alterations from beat to beat are translated into adaptive responses through intrinsic regulatory mechanisms^30^. Specifically, myocytes elongate in response to increased diastolic stretch by adding sarcomeres, and thicken under sustained systolic stress by incorporating filaments in parallel^31^. The Frank-Starling mechanism, a length-dependent activation of cardiac contraction, enables the heart to adjust its output according to changes in preload^32^.

The vascular system is continuously subjected to biomechanical forces arising from hemodynamic flow and pressure variations. Within this mechanical microenvironment, endothelial cells primarily perceive shear stress, whereas vascular smooth muscle cells (VSMCs) react to cyclic stretch induced by blood pressure fluctuations^33^. Hemodynamic shear stress appears in two primary forms: laminar shear stress (LSS), which promotes endothelial health and helps maintain vascular homeostasis^33^, and oscillatory shear stress (OSS), which plays a role in endothelial dysfunction and the development of atherosclerosis^34^. In response to these mechanical stimuli, endothelial cells modulate VSMC activity regarding relaxation and contraction through the secretion of paracrine factors such as nitric oxide (NO), Ca^2+^, oxidative stress mediators, angiotensin II (Ang II), and endothelin. VSMCs, in turn, regulate vascular tone, matrix synthesis, and degradation, thereby remodeling the vessel wall and maintaining its integrity and elasticity^33^.

Mechano-regulation in the cardiovascular system is mediated through specialized mechanosensors. For instance, integrins mediate mechanical stress-induced cardiac cytoskeletal remodeling^35^, while Piezo1 and TRP channels translate mechanical stretching into Ca^2+^-mediated signaling events, collaboratively regulating cardiac mechanotransduction and pathophysiological responses^36^-^38^.

## 3. Mechanical Stress and Myocardial Remodeling

**Figure 1:**
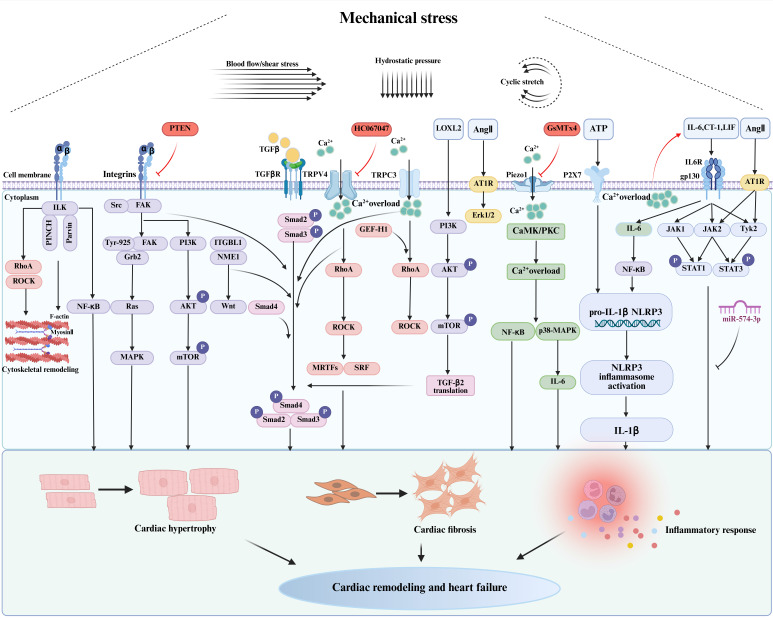
Regulatory mechanism of myocardial hypertrophy, fibrosis, and inflammatory response caused by mechanical stress. ILK: integrin linked kinase; RhoA: ras homolog family member A; ROCK: Rho-associated coiled-coil-containing protein kinase; NF-κB: nuclear factor-kappa B; FAK: focal adhesion kinase; MAPK: mitogen-activated protein kinase; PI3K: phosphatidylinositol 3-kinase; AKT: protein kinase B; mTOR: mechanistic target of rapamycin complex 1; ITGBL1: integrin beta-like 1; NME1: NME/NM23 nucleoside diphosphate kinase 1; TGFβ: transforming growth factor-beta; TGFβR: transforming growth factor-beta receptor; TRPV4: transient receptor potential vanilloid 4; MRTF: myocardin-related transcription factors; SRF: serum response factor; GEF-H1: the microtubule-associated Rho guanine nucleotide exchange factor; LOXL2: lysyl oxidase like 2; Ang Ⅱ: angiotensin II; AT1R: angiotensin II type 1 receptor; Erk1/2: extracellular signal-regulated kinase 1/2; CaMK: calmodulin-dependent protein kinase;PKC: protein kinase C; IL-6: interleukin-6; P2X7: P2X purinoceptor 7; ATP: adenosine triphosphate; IL-1β: interleukin-1β; NLRP3: nucleotide-binding and oligomerization domain (NOD)-like receptor thermal protein domain-associated protein 3; CT-1: cardiotrophin-1, LIF: leukemia inhibitory factor; JAK: janus kinase; Tyk2: tyrosine kinase 2; STAT: signal transducers and activators of transcription. Created in BioRender. gh, m. (2025) https://BioRender.com/3tn640y.

**Figure 2:**
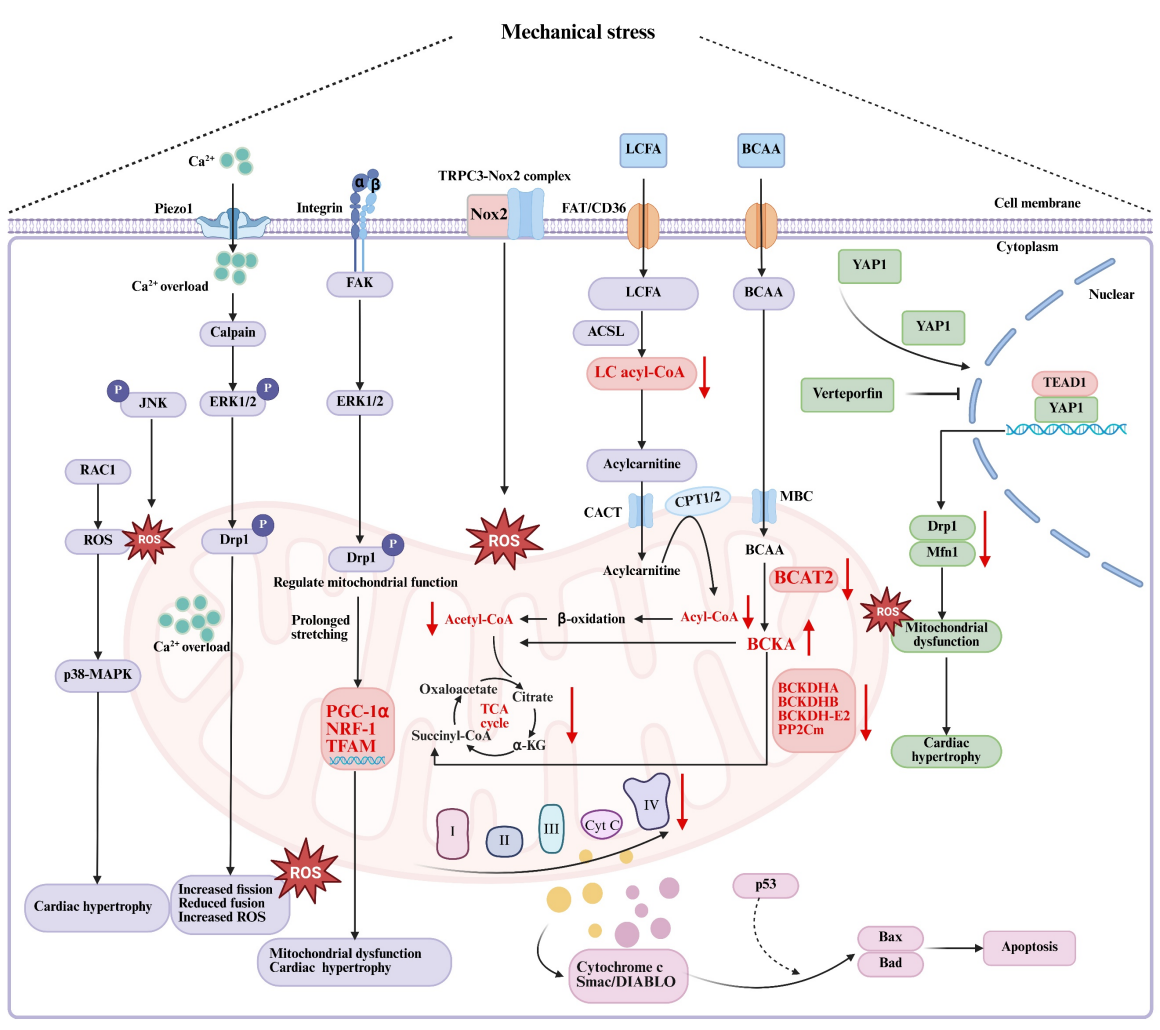
The regulatory mechanism of energy metabolism disorder caused by mechanical stress. RAC1: Rho family small GTPase 1; ROS: reactive oxygen species; MAPK: mitogen-activated protein kinase; JNK: c-Jun N-terminal kinase; Erk1/2: extracellular signal-regulated kinase 1/2; Drp1: dynamin-related protein 1; Mfn1: mitochondrial fusion protein 1; PGC-1α: peroxisome proliferator-activated receptor-γ coactivator-1α; NRF-1: nuclear respiratory factor; TFAM: mitochondrial transcription factor A; Nox2: NADPH oxidase 2; TRPC3: transient receptor potential canonical 3; LCFA: long-chain fatty acids; FAT: fatty acid transferase; ACSL: cardiac-specific acyl coenzyme A synthetase; CoA: coenzyme A; CACT: carnitine acylcarnitine translocase; CPT1/2: carnitine palmitoyltransferase1/2; BCAA: branched-chain amino acid; BCAT2: branched chain amino acid transaminase 2; BCKA: branched-chain α-keto acids; BCKDHA: branched chain α-ketoacid dehydrogenase (BCKD) subunit E1α; BCKDHB: branched chain α-ketoacid dehydrogenase subunit E1β; BCKDH-E2: branched chain α-ketoacid dehydrogenase subunit E2; PP2Cm: BCKD phosphatase; YAP1: Yes-associated protein 1. Created in BioRender. gh, m. (2025) https://BioRender.com/vp2v6ej.

Pathological cardiac hypertrophy, fibrosis, inflammation, endothelial dysfunction, metabolic reprogramming, and Ca^2+^ homeostasis imbalance are recognized as key pathological features of HFpEF. Mechanical stress acts as a pivotal factor in these pathological processes^39^, ^40^ (Figure 1 and Figure 2).

### 3.1 Myocardial response to mechanical stress

HFpEF is predominantly characterized by impaired left ventricular diastolic function, as demonstrated by diminished myocardial active relaxation and enhanced ventricular stiffness. These pathological changes are influenced by ECM components and intrinsic cardiomyocyte properties^41^. Although increased passive stiffness is commonly attributed to myocardial fibrosis, the most extensive HFpEF endocardial biopsy study to date identified moderate to severe fibrosis in only 27% of patients^42^, suggesting that additional factors beyond fibrosis contribute to ventricular wall stiffness in HFpEF. Post-translational modifications of titin, a protein essential for mechanical activity, have been recognized as a crucial mechanism for dynamically regulating diastolic passive tension. Thus, it can be deduced that ventricular wall stiffness comprises mechanical force components, while protein kinase A (PKA) can significantly mitigate passive tension^43^.

Mechanical stress, generated by cardiac contractions, is transmitted through the myocyte fiber architecture and the ECM, with mechanotransduction effectors responding to drive the biological effects of forces^9^. Initially, mechanotransduction and its downstream effects function as adaptive compensatory responses to increased mechanical load^40^. However, chronic elevation of mechanical stress induces pathological cardiac hypertrophy, a key feature of remodeling intended to accommodate the heightened load. Although this adaptive mechanism temporarily alleviates ventricular wall stress and maintains cardiac performance, hypertrophy is considered a pathological remodeling process when the myocardium becomes structurally and functionally compromised, with alterations occurring in both the cardiomyocyte and the ECM in response to the increased load^9^, ^44^.

In volume overload-induced eccentric hypertrophy, such as occurs in cases of mitral insufficiency, cardiomyocytes adapt to elevated diastolic pressure and volume through serial sarcomere addition, leading to enhanced longitudinal myocyte elongation and ventricular chamber dilation. This adaptation results in an increase in systolic wall stress, which in turn promotes wall thickening^44^. Conversely, conditions of pressure overload, such as HFpEF, arterial hypertension, or aortic stenosis, typically induce concentric hypertrophy through parallel sarcomere replication and transverse myocyte growth, culminating in ventricular wall thickening^44^, ^45^. These distinct remodeling patterns carry significant clinical implications, with eccentric hypertrophy often evolving into HFrEF and concentric hypertrophy predominantly associated with HFpEF^46^. Pathological hypertrophy enhances left ventricular mass and alters the myocardium's biomechanical properties, thus reducing myocardial compliance, impairing diastolic filling, significantly increasing left ventricular end-diastolic pressure (LVEDP), and contributing to the progression of HFpEF^47^.

*In vitro* investigations have established that mechanical stress is essential for the integrity of the myofibrillar structure in cultured cardiomyocytes. This stimulation may be derived from intrinsic forces due to phasic myocyte contraction or from extrinsic stress imparted by static substrate stretching^9^. To more faithfully replicate the three-dimensional (3-D) mechanical milieu encountered *in vivo*, Chen-Izu *et al.* engineered a cell-in-gel system in which individual cardiomyocytes are encapsulated within a viscoelastic hydrogel matrix. Within this configuration, the contraction of myocytes exerts mechanical forces on the surrounding gel. Stress analysis has shown that cardiomyocytes within this system are subjected to multi-axial loading, which includes longitudinal tension (along the long axis), transverse compression (along the short axis), and surface traction forces. Owing to isovolumetric constraints, contracting cardiomyocytes concurrently apply longitudinal pulling and transverse pushing forces against the gel. Consequently, both active contraction and external stretching impose stress on mechanosensors localized intracellularly and on the surface within this gel environment^48^. Simpson *et al.* conducted a systematic assessment of how varying degrees (0.5%, 1.0%, 2.5%, 5.0%, and 10.0%) and directions of mechanical stretch influence contractile protein turnover and accumulation in cultured neonatal rat cardiomyocytes (NRCMs). Compared with unstretched controls, contractile protein turnover was diminished by 50% to 100% in NRCMs subjected to 1.0% to 5.0% stretch, but remained unaltered at 0.5% or 10.0% stretch. The post-translational metabolism of myosin heavy chain and actin was regulated concomitantly with the total contractile protein pool. Moderate static stretch applied along the short axis of aligned cardiomyocytes in pulse-chase experiments has been shown to regulate myofibrillar protein accumulation, contractile protein turnover, and sarcomere structure. In contrast, stretching along the long axis resulted in no significant changes^49^. These findings suggest that specific directions of mechanical stimulation may govern the accumulation and post-translational metabolism of contractile proteins in cardiomyocytes^49^. This directional dependency was further supported by Gopalan *et al.*, who demonstrated that transverse stretch has a more profound impact than longitudinal stretch on sarcomere organization, hypertrophic growth, and cell-cell junction remodeling in cardiomyocytes^50^. Additionally, Yamamoto *et al.* provided evidence that cardiomyocytes possess the capability to discern whether mechanical stretch varies during diastole, systole, or both^51^, and activate distinct signaling pathways depending on the timing of mechanical stimulation within the cardiac cycle^49^. Collectively, these studies advance our understanding of the inherent heterogeneity in cardiomyocyte responses to mechanical stimuli.

### 3.2 Mechanical stress and cardiac fibrosis

Myocardial fibrosis represents a critical pathological aspect of cardiac remodeling in HFpEF and other cardiovascular diseases^52^. Mechanical stress is a fundamental driver of this fibrotic process. Under normal physiological conditions, cardiac fibroblasts, which are primarily responsible for ECM production in the myocardium, remain quiescent, thus maintaining ECM homeostasis. However, persistent or excessive mechanical stress initiates a pathological cascade that activates these fibroblasts, transforming them into α-smooth muscle actin (α-SMA)-expressing myofibroblasts with enhanced ECM synthetic capabilities^53^. This sustained overproduction of ECM leads to progressive ventricular stiffening and impaired diastolic function, ultimately contributing to the progression of heart failure^54^.

To explore the effects of varying modes of mechanical stretch on fibrosis, Husse *et al.* subjected adult rat ventricular fibroblasts to cyclical mechanical stretching (0.33 Hz) at three different elongations (3%, 6%, and 9%). The study demonstrated that a 9% cyclical stretch significantly increased the mRNA expression levels of collagen type I alpha 1 (Col1a), collagen type III alpha 1 (Col3a), matrix metalloproteinase-2 (MMP-2), and tissue inhibitor of MMP-2 (TIMP-2)^55^. These results indicate that hyper-stretching induces a fibrotic response, promoting ECM remodeling and contributing to heart failure progression^55^. Further investigations into mechanosensitive transcriptional regulation of specific ECM genes revealed a dependence on substrate stiffness. *In vitro* experiments demonstrated that cardiac fibroblasts cultured on hyaluronic acid substrates with Young's moduli of 3 kPa and 8 kPa showed significantly larger cell areas at and above 8 kPa than at 3 kPa, suggesting that the production of ECM in response to mechanical stretch is dependent on the stiffness of the matrix^56^. Excessive mechanical stretch also stimulates cardiac fibroblasts to produce profibrotic mediators, which may serve as critical fibrotic support factors in heart failure. Gan *et al.* found that culturing macrophages with conditioned medium from hyper-stretched wild-type mouse cardiac fibroblasts significantly enhanced the mRNA expression of profibrotic mediators, such as transforming growth factor-beta (TGF-β), interleukin-1β (IL-1β), and interleukin-13 (IL-13), whereas glucocorticoid regulated kinase 1 (SGK1) deficiency suppressed these effects^57^. An *in vivo* study further explored the relationship between mechanical stretch, cardiomyocyte stiffness, and cardiac diastolic function. Cardiomyocytes isolated from mice fed a high-fat/high-sugar diet (HFSD) and subjected to mechanical stretch exhibited significantly increased stiffness (stress/strain) *in vitro*, correlating with diastolic dysfunction (E/e') observed *in vivo*. During this process, mechanical stretch prolonged the Ca^2+^ transient decay in HFSD cardiomyocytes, and with cardiac pacing, the increase in stiffness and elevated diastolic Ca^2+^ levels diminished. These findings underscore the disruptive effects of mechanical stretch on myocardial Ca^2+^ homeostasis, leading to increased cardiomyocyte stiffening and diastolic dysfunction^58^.

Mechanical stress induces the activation of cardiac fibroblasts and contributes to the progression of myocardial fibrosis through a complex network of signaling pathways. Among these, the TGF-β signaling pathway plays a crucial role as a pivotal regulator^59^ (Figure 1). The TGF-β isoforms, comprising TGF-β1, TGF-β2, and TGF-β3, designate TGF-β1 as the principal mediator of myocardial fibrosis^60^. TGF-β is initially synthesized as an inactive precursor composed of mature TGF-β non-covalently linked to its latency-associated peptide (LAP). This complex additionally associates with latent TGF-β binding protein 1 (LTBP-1) within the ECM^61^. The liberation of TGF-β1 occurs via integrin-mediated mechano-activation. The Arg-Gly-Asp (RGD) peptides in LAP act as ligands for αv integrins^62^, which undergo conformational changes under mechanical stress, thus releasing active TGF-β^63^. This process facilitates prompt TGF-β activation in response to biomechanical stress. The released TGF-β initiates canonical SMAD-dependent signaling, where phosphorylated SMADs translocate to the nucleus to regulate the expression of fibrotic genes^64^. Additionally, it triggers transcription through noncanonical pathways, including p38 mitogen-activated protein kinase (p38 MAPK), extracellular regulated protein kinases (ERK), transforming growth factor-β-activated kinase 1 (TAK1), c-Jun N-terminal kinase (JNK), and Ras homolog family member A (RhoA) GTPase^65^. These pathways collectively drive fibroblast activation and ECM production.

ECM organization is critically regulated by cross-linking enzymes, notably lysyl oxidase (LOX) and transglutaminase 2 (TG2). In response to mechanical stress, cardiac tissues exhibit a significant upregulation of LOX and LOXL enzyme expression, with their enzymatic activities strongly correlating with fibrotic deposition, diastolic impairment, and progressive heart failure^66^, ^67^. In the transverse aortic constriction (TAC) model, Yang *et al.* demonstrated that LOXL2 is upregulated in the interstitium and modulates TGF-β2 translation through the phosphoinositide 3-kinase (PI3K)/protein kinase B (AKT)/mechanistic target of rapamycin complex 1 (mTORC1) signaling pathway. This molecular mechanism enhances TGF-β pathway activity, promoting fibroblast-to-myofibroblast transdifferentiation and the subsequent increase in expression of α-SMA, Col1a, and Col3a, which ultimately exacerbates heart failure (Figure 1). Conversely, both pharmacological inhibition and genetic ablation of LOXL2 have proven effective in mitigating pressure overload-induced myocardial fibrosis and improving ventricular performance. Notably, in HFpEF patients, cardiac LOXL2 levels correlate with the extent of cross-linked collagen, severity of diastolic relaxation abnormalities (E/e' ratio), and elevation of LVEDP^66^. Thus, targeting LOXL2 could represent a promising therapeutic approach for HFpEF.

MMPs and TIMPs are essential in the degradation and remodeling of the ECM. MMPs, a family of proteolytic enzymes, degrade various ECM components, contributing to the dynamic equilibrium and structural remodeling of the ECM. TIMPs, acting as specific inhibitors of MMPs, regulate their enzymatic activity, thus maintaining ECM homeostasis^68^. Disruption in the balance between MMPs and TIMPs, as occurs under conditions of mechanical stress overload, leads to altered gene expression of MMP2 and TIMP2, exemplified by the mechanical stretching of cardiac fibroblasts^69^. *In vitro* studies have shown that TGF-β induces a SMAD3-dependent matrix-preserving phenotype in cardiac fibroblasts, which suppresses the synthesis of MMP3 and MMP8 while enhancing the expression of TIMP1^68^. Initially, increased MMP activity promotes ECM degradation and angiogenesis, which are crucial for myocardial repair. However, as fibrosis advances, TIMP expression increases, resulting in decreased MMP activity and reduced ECM degradation. This imbalance leads to excessive ECM accumulation within the myocardial interstitium, exacerbating fibrosis^68^.

### 3.3 Mechanical stress and inflammatory response

Elevated mechanical stress not only inflicts direct structural damage on cardiac myocytes but also induces secondary pathological effects through the release of pro-inflammatory mediators^70^. The inflammatory process is recognized as a pivotal mechanism in the pathogenesis of HFpEF^71^. Under mechanical stress, both cardiomyocytes and cardiac fibroblasts secrete various inflammatory mediators, including interleukin-6 (IL-6), IL-1β, tumor necrosis factor α (TNF-α), and nucleotide-binding and oligomerization domain (NOD)-like receptor thermal protein domain-associated protein 3 (NLRP3)^72^, ^73^ (Figure 1). These mediators not only facilitate the recruitment of inflammatory cells such as macrophages and monocytes into the myocardial tissue but also trigger myocardial inflammatory responses. Additionally, they activate fibroblasts and endothelial cells through paracrine and autocrine signaling, which contributes to myocardial fibrosis and perpetuates a deleterious cycle^72^, ^74^.

IL-6 functions as a pivotal signaling cytokine that regulates inflammatory and immune responses^75^. Clinical investigations have demonstrated that patients with HFpEF exhibit significantly elevated levels of circulating IL-6, which are strongly correlated with the severity of the disease, functional impairments, and diminished exercise capacity^76^. Palmieri *et al.* observed that myocardial IL-6 mRNA and protein levels rise in response to varying degrees of mechanical stretch stimulation in normal rats; notably, moderate and severe acute stretch result in a substantial increase in these levels^73^. This induction process involves mechanical stretch leading to an elevation in intracellular Ca^2+^ concentrations in neonatal rat and murine cardiomyocytes, which subsequently upregulates the expression of IL-6, cardiotrophin-1 (CT-1), leukemia inhibitory factor (LIF), and Ang II. These changes activate glycoprotein 130 (gp130), and LIF and CT-1 then activate Janus kinase 1 (JAK1), JAK2, tyrosine kinase 2 (Tyk2), signal transducers and activators of transcription 1 (STAT1), and STAT3. Ang II activates the JAK2, Tyk2, STAT1, and STAT3 signaling pathways^77^, which promote myocardial hypertrophy, myocardial interstitial fibrosis, and an inflammatory cardiac response^78^. Aerobic exercise mitigates these pathological alterations through the upregulation of myocardial expression of miR-574-3p^78^. Furthermore, IL-6 enhances the synthesis of C-reactive protein (CRP) induced by mechanical stretch via the stretch-activated channel nuclear factor-kappa B (NF-κB) signaling pathway, thereby promoting vascular inflammatory responses^79^. Mechanical stress also activates the NF-κB and the MAPK signaling pathways in cardiac tissues, thereby forming a complex interaction network that cooperatively regulates inflammation and fibrosis responses. The NF-κB pathway primarily mediates the initiation and maintenance of inflammatory responses in cardiac myocytes, whereas the MAPK pathway plays a more extensive role in regulating cellular phenotypic transformation and ECM homeostasis in cardiac fibroblasts. There is intricate crosstalk between these pathways; for example, the MAPK pathway enhances the transcriptional activity of NF-κB by promoting its nuclear translocation, while NF-κB upregulates the expression of genes associated with the MAPK pathway, thus establishing a positive feedback loop^80^-^82^. This feedback mechanism potentially perpetuates a vicious cycle of inflammatory and fibrotic cascades, thus accelerating the progression of cardiovascular diseases.

Activation of NF-κB leads to the upregulation of NLRP3, a cytosolic immune sensor that forms signaling complexes in response to a variety of pathological stimuli, including metabolic disturbances, mitochondrial dysfunction, aging, and environmental factors. This activation facilitates the maturation of the pro-inflammatory cytokines IL-1β and interleukin-18 (IL-18), thereby promoting inflammatory responses and cell death^83^. Higashikuni *et al.* demonstrated that, under conditions of pressure overload, activation of the NLRP3 inflammasome through heart-brain interactions precipitates the maturation and release of myocardial IL-1β, exacerbating cardiac inflammatory responses and contributing to cardiac hypertrophy and fibrosis^72^. Furthermore, the pharmacological inhibition of extracellular ATP release from sympathetic nerve terminals, or the genetic knockout of P2X purinoceptor 7 (P2X7), has been shown to attenuate NLRP3 inflammasome activation in the myocardium. These findings indicate that the ATP/P2X7 signaling axis plays a crucial role in ameliorating pressure overload-induced cardiac inflammation and hypertrophy (Figure 1), underscoring the therapeutic potential of targeting neuro-cardiac signaling pathways in mechanical stress-related cardiomyopathy^72^.

### 3.4 Mechanical stress and endothelial dysfunction

The vascular endothelium, a continuous monolayer of cells lining the inner surface of blood vessels, serves as both a structural barrier separating the blood from the vessel wall and as a dynamic functional interface^84^. It is indispensable for maintaining vascular homeostasis, regulating hemodynamics, controlling vascular permeability, and preventing the adhesion and aggregation of platelets and leukocytes^85^. In its healthy state, the endothelium dynamically modulates vascular tone by releasing a balanced array of vasodilators and vasoconstrictors in response to various mechanical and biochemical stimuli^86^.

Endothelial dysfunction constitutes a critical pathophysiological mechanism in HFpEF. Comorbidities such as hypertension, diabetes mellitus, and obesity instigate a state of microvascular endothelial inflammation. This inflammation is mediated by increased oxidative stress, uncoupling of endothelial NO synthase (eNOS), reduced bioavailability of NO, and impaired signaling through cyclic guanosine monophosphate (cGMP) and protein kinase G (PKG). These factors collectively lead to endothelial dysfunction within the coronary microcirculation^87^, ^88^. Additionally, the regulation of both collagen turnover and titin homeostasis is critically reliant upon cGMP/PKG signaling, which plays an essential role in modulating the passive mechanical properties of the left ventricle, particularly myocardial stiffness^5^, ^89^. Kishimoto *et al.* demonstrated that flow-mediated vasodilation and nitroglycerin-induced vasodilation are significantly reduced in patients with HFpEF compared to those without heart failure. Additionally, these patients exhibit a notable increase in brachial artery intima-media thickness and brachial-ankle pulse wave velocity, indicating more pronounced vascular alterations^90^. Clinical investigations have established that an impaired coronary microcirculation reserve is independently associated with increased hospitalizations due to heart failure. Remarkably, patients presenting with both diastolic dysfunction and a compromised coronary microcirculation reserve at baseline are at a fivefold increased risk of hospitalization for heart failure^91^. These findings are corroborated by studies conducted on leptin-resistant, obese, hypertensive ZSF1 rats, which develop HFpEF, underscoring that myocardial endothelial dysfunction plays a significant role in the pathogenesis of HFpEF^91^.

The endothelial lining of blood vessels is continuously exposed to mechanical forces generated by blood flow. These forces, known as shear stress, are detected by endothelial cells, triggering intracellular signaling cascades that are essential for regulating vascular physiology^92^. In HFpEF, impaired diastolic function of the left ventricle results in elevated filling pressures, which increase both vascular wall stress and myocardial cellular mechanical stress. Concurrently, diminished cardiac performance leads to changes in systemic hemodynamics. These alterations disrupt the physiological shear stress exerted on vascular endothelial cells and contribute to the progression of endothelial dysfunction^93^, ^94^. This abnormal mechanical environment induces morphological changes in the endocardial endothelium, including hypertrophy of the synthetic apparatus and cytoskeletal reorganization, culminating in cardiac dysfunction^95^. Among the comorbidities frequently associated with HFpEF, a study by Felaco *et al.* found that diabetes does not significantly alter the transcription or translation levels of eNOS in rat myocardial tissue. However, analyses of both the endothelium and vascular wall reveal a decrease in eNOS immunoreactivity and diminished nicotinamide adenine dinucleotide phosphate (NADPH) reactivity in the diabetic group compared to non-diabetic controls^96^. Similar findings have been reported in experimental models that incorporate both hypertension and diabetes^97^. Furthermore, hyperglycemia intensifies this damage by promoting the overproduction of mitochondrial reactive oxygen species (ROS), which leads to oxidative damage to mitochondrial DNA^98^. The nuclear translocation of damaged mitochondrial DNA activates the poly (ADP-ribose) polymerase 1 (PARP-1) pathway, resulting in inhibition of glyceraldehyde-3-phosphate dehydrogenase (GAPDH) activity, disruption of glycolysis^99^, and marked upregulation of the p38 MAPK-cFOS inflammatory axis, which collectively promote endothelial inflammation^100^. In the pathological milieu associated with HFpEF, reduced eNOS expression, along with elevated levels of ROS and inflammation, points to a state of endothelial dysfunction. This dysfunction is a consequence of disease-induced alterations in the vascular mechanical environment, leading to a cascading sequence of pathological responses that exacerbate cardiac impairment.

### 3.5 Mechanical stress and mitochondrial energy metabolism

As the primary circulatory pump, the heart depends on cardiomyocytes to generate substantial quantities of ATP, essential for sustaining its continuous and coordinated systolic and diastolic functions^101^. Cardiac energy metabolism is primarily fueled by mitochondrial oxidative phosphorylation, which under basal conditions, derives 70% to 90% of its ATP from long-chain fatty acid β-oxidation. The remainder of the ATP is produced from glucose, lactate, and minor contributions from alternative substrates such as ketones and amino acids^102^, ^103^. Within the mitochondria, mitochondrial creatine kinase facilitates the transfer of the high-energy phosphate group from ATP to creatine, producing phosphocreatine (PCr). This molecule then diffuses across the mitochondrial membrane into the cytosol, where cytosolic creatine kinase catalyzes the regeneration of ATP from ADP. This sophisticated mechanism allows cardiomyocytes to utilize ATP efficiently for both contraction and relaxation, thereby supporting the heart's energy-intensive activities^101^, ^103^.

Mechanical stress on the heart exhibits biphasic effects on mitochondrial regulation. Physiological levels of mechanical stimulation are crucial for maintaining mitochondrial homeostasis, whereas pathological stress impairs mitochondrial quality control mechanisms and diminishes mitochondrial functionality^21^, ^104^. In individuals with heart failure, as well as in murine models of pressure overload-induced cardiac dysfunction, impaired mitochondrial oxidative energy metabolism contributes to decreased levels of long-chain acyl-coenzyme A (CoA), which accelerates cardiac remodeling and the progression of heart failure. Cardiac-specific overexpression of acyl-CoA synthetase-1 (ACSL1) in mice has been shown to mitigate pressure overload-induced cardiac dysfunction by reducing toxic lipid accumulation, restoring long-chain acyl-CoA levels, and enhancing mitochondrial bioenergetics. Additionally, mechanical assist devices have proven effective in restoring long-chain acyl-CoA levels in heart failure patients^105^. Impaired catabolism of branched-chain amino acids (BCAAs) due to downregulated expression of key enzymes such as branched-chain amino acid transaminase 2 (BCAT2), branched-chain α-ketoacid dehydrogenase (BCKD) subunits (BCKDHA, BCKDHB, and BCKDH-E2), and BCKD phosphatase PP2Cm, leads to pathological accumulation of branched-chain α-keto acids (BCKAs). This accumulation compromises mitochondrial complex I activity, exacerbates oxidative stress, and fosters maladaptive cardiac remodeling. However, pharmacological activation of BCKA dehydrogenase has been demonstrated to counteract this pathological process effectively^106^ (Figure 2).

Mitochondria serve as the principal hub of energy metabolism within cardiomyocytes, directing the oxidation and catabolism of various substrates to produce ATP. Mitochondrial dysfunction plays a critical role in the development of energetic impairment, a defining feature of heart failure pathogenesis. The chronic pressure overload model, induced by abdominal aorta banding, leads to left ventricular hypertrophy, diminished maximal oxidative capacity, reduced activity of mitochondrial respiratory chain complex IV, increased mitochondrial H_2_O_2_ production, free radical leak, and citrate synthase activity in the subendocardium and subepicardium^107^. *In vitro* studies employing a sustained mechanical stretch model reveal that mechanical strain prompts mitochondria-mediated apoptosis in cardiomyocytes. This process is marked by the release of cytochrome c and Smac/DIABLO from the mitochondria into the cytoplasm, alongside a decrease in mitochondrial membrane potential (∆ψm), which signifies the opening of the mitochondrial permeability transition pore (PTP). The opening of the PTP facilitates the upregulation of pro-apoptotic members of the Bcl-2 family, such as Bax and Bad, thereby inducing apoptosis. Additionally, p53 is implicated in playing a partially mediating role in the apoptosis induced by mechanical stretch. The inhibition of this apoptotic pathway by the PTP inhibitor Cyclosporin A is also noted^108^ (Figure 2).

The proteins involved in mitochondrial fission and fusion operate within a complex signaling network to maintain mitochondrial homeostasis and fulfill pivotal roles in modulating cardiac responses to mechanical stress, including ischemia-reperfusion injury, heart failure, and cardiomyopathy. Dynamin-related protein 1 (Drp1) has been shown to play a central role in the regulation of mitochondrial fission and autophagy in the myocardium^109^. Under normal physiological conditions, Drp1 is predominantly localized in the cytosol but is swiftly recruited to mitochondria upon mechanical stimulation. This translocation initiates mitochondrial shortening and inhibits mitochondrial autophagy during the 2-5 days following TAC, eventually leading to profound bioenergetic compromise and heart failure^110^. Moreover, cardiac-specific ablation of Drp1 impairs mitochondrial degradation, resulting in the accumulation of pathologically enlarged and vacuolar mitochondria, compromised oxidative phosphorylation, and lethal cardiomyopathy in mice^111^. Similarly, the genetic deletion of mitochondrial fusion proteins Mfn1 and Mfn2 in murine models induces abnormal mitochondrial fragmentation and structural disorganization, consequently causing ventricular wall thickening, increased cardiac mass, and the onset of eccentric hypertrophy^112^. Additionally, pathological mechanical stress disrupts mTORC1 signaling pathways, which impedes mitophagy and leads to the accumulation of dysfunctional mitochondria, further exacerbating cardiac dysfunction^113^. The partial pharmacological inhibition of mTORC1 has been shown to alleviate cardiac remodeling and heart failure induced by pressure overload^114^. Recent research by Yue *et al.* revealed that mechanical stress activates the mechanosensitive transcriptional cofactor Yes-associated protein 1 (YAP1), which, upon binding to the TEA domain transcription factor 1 (TEAD1) motif, transcriptionally represses the expression of pivotal mitochondrial dynamic regulators such as Drp1 and Mfn1. This repression disrupts mitochondrial ultrastructure and function, accompanied by increased ROS production, DNA damage, and inhibition of cardiomyocyte proliferation^115^ (Figure 2). Collectively, these findings underscore the YAP1 pathway as a pivotal mediator of stress-induced metabolic dysregulation and structural remodeling, suggesting that its pharmacological modulation might hold therapeutic promise for the management of HFpEF.

Under physiological conditions, the mitochondrial electron transport chain effectively couples more than 98% of electron flow to ATP synthesis, with a mere 1% to 2% contributing to the generation of mitochondrial ROS (mtROS)^116^. At basal levels, mtROS serve as vital signaling molecules that orchestrate a variety of critical cellular functions, including cell differentiation, programmed cell death, metabolic homeostasis, and immune modulation^117^. However, excessive mtROS production induces oxidative stress, which plays a substantial role in HFpEF development and progression^118^. Research conducted by Iribe *et al.* indicated that mechanical stretch in cardiac tissue activates the electron transport chain, leading to hyperpolarization of the mitochondrial membrane potential (Δψm) and subsequently triggering NADPH oxidase (NOX)-mediated overproduction of ROS^119^. *In vitro* studies have demonstrated that mechanical stretch results in the phosphorylation of ERK1/2, while high-amplitude mechanical stretch further activates the JNK signaling pathway. The concurrent activation of both ERK1/2 and JNK pathways correlates with elevated ROS levels, which in turn promote cardiomyocyte apoptosis and cardiac hypertrophy^120^. Similarly, *in vivo* experiments showed that excessive mechanical stress initiates a Rho family small GTPase 1 (RAC1)-mediated redox cascade, leading to a progressive accumulation of ROS in rat ventricular cardiomyocytes. This cascade facilitates apoptotic signaling and activates the downstream p38 MAPK pathway, contributing significantly to the development of pathological cardiac hypertrophy^121^. Zhou *et al.* have identified that the nuclear factor erythroid 2-related factor 2 (Nrf2) signaling pathway acts as the primary intracellular antioxidant defense mechanism, regulating the expression of antioxidant and detoxification enzymes, thereby effectively mitigating ROS and shielding cells from oxidative damage^122^. Nevertheless, pathological mechanical stress diminishes the activity of the Nrf2 signaling pathway, compromising antioxidant capacity and exacerbating oxidative stress and myocardial injury^122^, ^123^. Furthermore, neutrophil-derived myeloperoxidase (MPO) exacerbates oxidative stress by producing potent oxidants such as hypochlorous acid, thus intensifying oxidative damage and inflammatory responses^124^. These insights have led to the clinical exploration of the MPO inhibitor mitiperstat (AZD4831) as a promising therapeutic agent for HFpEF, targeting oxidative stress^124^.

### 3.6 Mechanical stress and calcium imbalance

Ca^2+^ acts as a pivotal regulator in the excitation-contraction coupling of cardiomyocytes and serves as an essential second messenger in the control of metabolic activities, apoptotic pathways, and transcriptional regulation^125^. During physiological stretching, microtubule-mediated X-ROS signaling rapidly activates the reduced form of NOX2, leading to the production of ROS that trigger typical Ca^2+^ sparks, which are the primary Ca^2+^ release events in the myocardium^126^. However, under pathological conditions, abnormal mechanical stress disrupts cardiac Ca^2+^ homeostasis^48^, thereby enhancing Ca^2+^ influx through L-type Ca^2+^ channels and stretch-activated ion channels, as well as facilitating Ca^2+^ release from the sarcoplasmic reticulum via the ryanodine receptor (RyR). This process leads to a Ca^2+^ overload in cardiomyocytes^127^. Elevated intracellular Ca^2+^ concentrations subsequently augment signaling pathways involving protein kinase C (PKC), calcineurin, and Ca^2+^/calmodulin-dependent protein kinase II (CaMKII), which result in alterations in gene expression relevant to the pathology of cardiac diseases^125^.

Research has shown that abnormal mechanical stress activates mechanosensitive Ca^2+^ channels, such as Piezo1 and TRP channels, on the cardiomyocyte membrane, promoting excessive Ca^2+^ influx and cytoplasmic overload^128^. This type of mechanical stress impairs the function of RyRs and sarcoplasmic/endoplasmic reticulum Ca^2+^ ATPase 2a (SERCA2a) in sarcoplasmic reticulum Ca^2+^ channels. RyRs, which are the primary sarcoplasmic reticulum Ca^2+^ release channels, undergo pathological phosphorylation at the S2808 and S2814 sites due to the combined effects of mechanical stress, oxidative damage, and neurohormonal activation. This phosphorylation increases the probability of channel opening, leading to sarcoplasmic reticulum Ca^2+^ leakage, elevated cytoplasmic Ca^2+^ levels, diastolic dysfunction, arrhythmias, and cardiomyocyte apoptosis^129^, ^130^. SERCA2a is crucial for maintaining Ca^2+^ homeostasis by facilitating the reuptake of cytoplasmic Ca^2+^ into the sarcoplasmic reticulum, thereby reducing cytoplasmic Ca^2+^ concentration and promoting myocardial relaxation^131^. In the heart, mechanical stress, oxidative stress, and inflammatory responses impair SERCA2a function through a reduction in activity, decreased expression levels, or abnormal phosphorylation modifications. These alterations compromise the sarcoplasmic reticulum's Ca^2+^ reuptake capacity, leading to elevated cytoplasmic Ca^2+^ levels, prolonged Ca^2+^ transient duration, and exacerbating diastolic dysfunction^132^. The coordinated dysregulation of both RyR-mediated Ca^2+^ release and SERCA2a-dependent Ca^2+^ reuptake establishes a self-amplifying cycle of Ca^2+^ mishandling that propels the progression of HFpEF, with each component presenting potential therapeutic targets for intervention.

## 4. Mechanotransduction Effectors

Mechanical stress acts as a critical extracellular stimulus necessitating the transduction into intracellular biochemical signals through cellular mechanosensing apparatus to govern cellular function and maintain tissue homeostasis^104^. Mechanosensitive ion channels function as molecular force transducers that detect membrane tension perturbations directly, initiating rapid adaptive responses through ion flux-dependent modulation of gene expression and cytoskeletal reorganization^133^. In cardiac tissue, resident cardiomyocytes and fibroblasts express a phylogenetically conserved array of mechanoreceptors, including Piezo channels, integrins, and TRP channels. These mechanoreceptors translate mechanical stimuli into precisely regulated biochemical signaling cascades^22^, ^134^. This mechanochemical conversion triggers downstream effectors such as the YAP/TAZ pathway, MAPK signaling, and Ca^2+^-dependent processes, which ultimately control transcriptional reprogramming and phenotypic modulation through cytoskeletal rearrangement. Collectively, these processes determine the architecture and functional output of cardiac tissue^135^. Dysregulation of these mechano-adaptive processes is implicated in pathological cardiac remodeling observed in disease states (Figures 1 and 2).

### 4.1 Integrin cytoskeleton in mechanotransduction

#### 4.1.1 Integrin

Integrins represent a fundamental class of mechanotransducers that facilitate bidirectional mechanical communication across cell-ECM and cell-cell interfaces via sophisticated force-sensing mechanisms^136^. These integrin receptors operate as heterodimers consisting of α and β subunits, with mammalian systems expressing 18 α and 8 β subunits that together form 24 distinct integrin variants^137^. Each integrin molecule features a large extracellular domain responsible for ligand binding, a transmembrane region, and a short cytoplasmic tail^138^. The extracellular domain undergoes force-dependent conformational changes upon ECM engagement, while the cytoplasmic domains, characterized by conserved β subunit tails and divergent α subunit sequences, serve as platforms for mechano-signaling complex assembly^139^. Since integrins lack intrinsic enzymatic activity or actin-binding capabilities, they require adaptor proteins to convey ECM-derived mechanical signals to the intracellular milieu. These adaptors link integrin cytoplasmic domains either to the actin cytoskeleton or to downstream signaling components, initiating mechanotransduction cascades^136^. Integrin-linked kinase (ILK), focal adhesion kinase (FAK), vinculin, paxillin, talin, and α-actinin participate in this process as signaling intermediaries^22^. The precise mechanical decoding by this integrin-adaptor system enables cells to sense and respond adaptively to diverse ECM mechanical properties, particularly variations in stiffness, with exceptional real-time regulation.

Emerging evidence underscores the importance of integrin-mediated mechanotransduction in regulating cardiac pathophysiology under conditions of mechanical stress overload. Different integrin isoforms and their downstream effectors orchestrate cell-specific responses during the progression of disease. Burgess *et al.* examined the expression of integrin subunits, cellular migration behaviors, and gel contraction capacities in cardiac fibroblasts isolated from rats subjected to either a 10-week regimen of treadmill exercise or experimentally induced hypertension. Their findings indicated that the α1 and α2 integrin subunits were downregulated in the hypertensive group, whereas the α5 and β1 integrin subunits were upregulated^140^. Functional analyses disclosed that fibroblasts from both the exercised and hypertensive rats exhibited diminished migration on collagen compared to the control group. On fibronectin, fibroblasts from the exercised group migrated more rapidly, whereas those from the hypertensive group migrated more slowly than the controls. These results suggest that cardiac fibroblasts adapt to pathological mechanical stress by modulating integrin expression, thereby facilitating myocardial fibrosis and remodeling^140^.

Ding *et al.* reported an increase in β1 integrin accumulation on cardiomyocyte surfaces and within the ECM during the early stages of pressure overload-induced heart failure, positing that active integrin shedding may contribute to disease progression^141^. In the streptozotocin (STZ)-induced diabetes heart failure model, Talior-Volodarsky *et al.* noted a significant upregulation of α11 integrin in cardiac fibroblasts isolated from diabetic rats. Genetic suppression of both α11 integrin and TGF-β receptors led to an increased expression of TGF-β2 and α-SMA, while inhibition of Smad3 signaling arrested this process. These findings imply that α11 integrin and TGF-β2 may facilitate myofibroblast differentiation in cardiac fibroblasts in the diabetic heart, thus promoting the progression of cardiac fibrosis and heart failure^142^. *In vitro* studies further demonstrated that glycated collagen specifically upregulates collagen-binding α11 integrin through activation of the TGF-β/Smad2/3 pathway in isolated cardiac fibroblasts^143^. Nevertheless, in streptozotocin-induced type 1 diabetes, α11 integrin was observed to mediate not only myocardial fibrosis but also significant matrix-preserving actions that safeguard the heart against dysfunction^144^, thereby highlighting its dual role in mediating fibrotic remodeling and myocardial preservation during the progression of diabetic heart disease. Preclinical drug trials have shown that dapagliflozin can effectively treat diabetic cardiomyopathy by inhibiting the expression of mechanical signal proteins under varying ECM stiffness conditions. Inhibition of the Ang II type 1 receptor (AT_1_R)/FAK/NOX2 pathway reduced the production of reactive oxygen species in myocardial tissue, diminishing collagen deposition, myocardial injury, and oxidative stress, and ultimately delaying the progression of diabetic cardiomyopathy. Furthermore, dapagliflozin suppresses the expression of integrin β1 induced by ECM stimulation, though it exhibits no significant effect on Piezo1^145^.

FAK has been identified as a crucial mediator in the activation of cardiac fibroblasts under cyclic stretch, as well as in the progression of cardiac fibrosis and heart failure resulting from mechanical stress overload. Mechanical stretch initiates the phosphorylation of FAK at Tyr-397 via integrin-mediated mechanisms, thereby creating a high-affinity binding site for the SH2 domain of c-Src^146^. Subsequent recruitment of c-Src leads to the phosphorylation of Tyr-925 on FAK, establishing a Grb2 binding site that links FAK to the Ras/MAPK signaling pathway^147^ (Figure 1). As a result, mechanical stress activates MAPK, thereby promoting cardiac hypertrophy through the integrin-FAK-Src-Ras signaling pathway in cardiomyocytes^148^. Furthermore, FAK facilitates the activation of the PI3K/AKT/mTOR signaling pathway, which induces cardiomyocyte hypertrophy^149^ (Figure 1). The tumor suppressor PTEN inhibits the integrin-mediated outside-in signals^148^, while targeted silencing of FAK through specific small interfering RNA (siRNA) further diminishes fibrosis, decreases collagen content, suppresses MMP-2 activity, and reverses cardiac hypertrophy and failure induced by mechanical stress overload^150^.

FAK is vital in the mechanotransduction-regulated biogenesis of myocardial mitochondria. Cyclic mechanical stretch activates mitochondrial fission mediated by Drp1 through the FAK and ERK1/2 signaling pathways, enabling cardiomyocytes to metabolically adapt to ECM remodeling and mechanical stress via the FAK-ERK1/2-Drp1 axis^151^ (Figure 2). However, prolonged mechanical stretching increases the expression of core mitochondrial transcriptional regulators, including mitochondrial transcription factor A (TFAM), nuclear respiratory factor (NRF-1), and peroxisome proliferator-activated receptor-γ coactivator-1α (PGC-1α), which leads to enhanced mitochondrial biogenesis and concurrent pathological hypertrophy in cardiomyocytes^152^. Both *in vivo* and *in vitro* studies have shown that FAK silencing markedly reduces the upregulation of PGC-1α, NRF-1, mitochondrial DNA (mtDNA), and left ventricular hypertrophy caused by pressure overload^152^.

ILK is a pivotal regulator within integrin-mediated signaling pathways^153^. It forms a functional complex with PINCH, a LIM-domain protein, and Parvin, a protein that binds both actin and paxillin, known collectively as the ILK-PINCH-Parvin (IPP) complex. This complex is essential for the regulation of actin cytoskeleton dynamics^154^. *In vitro* experiments have shown that ILK activation promotes the nuclear translocation of NF-κB, thus enhancing the expression of genes for collagen I and connective tissue growth factor (CTGF) in cardiac fibroblasts under Ang II stimulation^155^. An *in vivo* study has indicated that cardiac-specific knockout of ILK impairs myocyte architecture, diminishes Akt-mediated cardiac protection, and accelerates the progression of cardiac fibrosis and heart failure^156^.

Integrin beta-like 1 (ITGBL1), an ECM protein structurally related to β-integrins. Chen *et al.* proved that ITGBL1 is upregulated in the heart in response to pressure overload, where it contributes to the progression of cardiac hypertrophy and heart failure. *In vitro* studies reveal that overexpression of ITGBL1 in neonatal rat cardiac fibroblasts (NRCFs) significantly increases the transcription of profibrotic genes includingα-SMA, Col1a, and Col3a. Additionally, it augments the expression of hypertrophic biomarkers, including atrial natriuretic peptide (ANP) and brain natriuretic peptide (BNP). Mechanistically, ITGBL1 interacts with nucleoside diphosphate kinase 1 (NME1), facilitating the activation of the TGF-β/Smad2/3 and Wnt signaling pathways. These pathways synergistically promote fibroblast activation and cardiomyocyte hypertrophy. In a murine TAC model, silencing ITGBL1 attenuates cardiac fibrosis and hypertrophic remodeling, concurrently enhancing cardiac function^157^. These findings suggest that ITGBL1 could serve as a viable therapeutic target for ameliorating heart failure prompted by mechanical stress overload.

#### 4.1.2 Cytoskeleton

The cytoskeleton, an essential intracellular structure, maintains cellular morphology and mechanical stress stability. Comprising primarily microfilaments (actin), microtubules, and intermediate filaments, the cytoskeleton forms a complex interconnected network. This network connects mechanoreceptors on the cell membrane to the intracellular milieu, transducing mechanical signals from the ECM and modulating cellular mechano-sensitivity through its tension, rigidity, and dynamic properties. In cardiomyocytes, dynamic remodeling of the cytoskeleton is crucial in regulating cell contraction, relaxation, and responses to mechanical stress^158^. Actin, the primary component of the cardiomyocyte cytoskeleton, undergoes dynamic assembly and disassembly, directly influencing contractile function. Under mechanical stress, actin filaments reorganize, altering cellular mechanical properties. For instance, when cardiomyocytes are subjected to tensile stress, actin filaments realign along the applied force's direction to enhance cellular tensile strength. This reorganization is regulated by proteins such as cofilin and profilin, which modulate actin polymerization and depolymerization, thereby affecting cytoskeletal stability^134^, ^159^. Microtubules and intermediate filaments provide structural support and stability to cardiomyocytes. Microtubules maintain cell morphology and facilitate intracellular transport through their dynamic assembly and disassembly, while intermediate filaments (desmin) enhance cytoskeletal stability by interacting with actin and microtubules^6^, ^160^.

Cytoskeletal proteins are activated in response to mechanical stimulation of cells or tissues. Tension within the cytoskeleton arises from dynamic interactions between the ECM and intercellular communication^134^. This tension facilitates the establishment of a network structure that extends from the ECM to detect extracellular signals and biophysical mechanics. Cell-cell interactions, mediated through adhesion junctions and desmosomes, enable the transmission of cytoskeletal tension into the cellular interior and the nucleus. Both ECM interactions and cell-cell connections regulate components of the cytoskeleton, encompassing intermediate filaments, microtubules, and actin, thereby modulating cellular mechanical properties and mechanisms of force transduction^134^. Prolonged cytoskeletal stress contributes to myocardial hypertrophy, which in turn drives structural and functional remodeling of actin, intermediate filaments, microtubules, and their associated networks.

Cytoskeletal remodeling is regulated by several signaling pathways, among which the integrin-mediated regulation of the RhoA/Rho-associated coiled-coil-containing protein kinase (RhoA/ROCK) pathway is pivotal (Figure 1). Activation of RhoA stimulates ROCK kinase, promoting actin polymerization and stress fiber formation, which enhances cellular contractility. However, persistent RhoA/ROCK signaling induces maladaptive remodeling, leading to cardiomyocyte hypertrophy and increased myocardial stiffness, thereby impairing diastolic compliance^161^. Additionally, the FAK and Src family kinases regulate cytoskeletal remodeling through modulation of integrin-cytoskeleton interactions^35^. Notably, the application of increasing force stretches structural proteins, and upon exceeding a critical threshold, activation domains are exposed. This exposure triggers the recruitment of talin to the β subunits of integrins, vinculin, paxillin, and FAK, generating a broad spectrum of intracellular signals^162^.

The cytoskeleton responds to mechanical stress overload by altering the architectural mechanics of cardiomyocytes, inducing cytoskeletal remodeling through collagen and titin mediation, which increases passive myocardial stiffness and contributes to the development of HFpEF^5^. Consequently, the mechanically regulated cytoskeletal network may represent a promising therapeutic target for ameliorating cellular stiffness in HFpEF. Moreover, the cardiomyocyte cytoskeleton network plays an essential role in maintaining cellular homeostasis. Post-translationally detyrosinated microtubules serve as a critical determinant of viscoelastic behavior in cardiomyocytes and are a primary factor influencing cardiac diastolic function. Caporizzo *et al.* demonstrated that in failing myocardium, stable and detyrosinated microtubules generate viscous forces during diastolic stretch, thereby increasing cardiomyocyte stiffness^6^. Suppressing microtubule detyrosination reduces the stiffness of failing cardiomyocytes and improves relaxation kinetics, suggesting potential for microtubule-targeted interventions in the treatment of patients with HFpEF^163^.

### 4.2 Piezo channels in mechanotransduction

Mechanosensitive ion channels (MICs) are a crucial class of mechanoreceptors that directly detect changes in mechanical stress across the cytomembrane, translating these variations into altered ion permeability and thereby modulating membrane potential and intracellular ion concentrations^164^. Embedded within the membrane, these mechano-receptors are pivotal in directing the activation of cardiac fibroblasts and the remodeling of fibrosis, encompassing the regulation of cell proliferation, the promotion of myofibroblast differentiation, the modulation of ECM remodeling, and the facilitation of paracrine signaling pathways^165^. Recent advancements in structural biology and electrophysiology have shed light on the molecular architecture governing MIC activation, demonstrating that force-induced conformational changes within channel domains, along with their dynamic interactions with cytoskeletal components, dictate the gating behavior in response to mechanical stress. This provides a comprehensive mechanistic framework for elucidating the conversion of physical forces into biochemical signals during cardiac remodeling processes^22^, ^134^, ^166^.

Piezo channels, a recently identified class of mechanosensitive cation channels that includes the Piezo1 and Piezo2 subtypes, are highly sensitive to mechanical stimuli. Piezo1 is extensively expressed throughout the cardiovascular system, playing a critical role in modulating vascular homeostasis, hemodynamics, and cardiac remodeling processes^167^. This channel responds to a variety of physical stimuli, including membrane deformation, tensile strain, shear stress, and hydrostatic pressure, all of which alter membrane tension^168^. Under mechanical stress, Piezo1 channels are distributed across the cell membrane, and the lipid interactions proximal to these channels form a dome-like configuration, enhancing their responsiveness to mechanical signals^169^. Upon activation, the influx of cations mediated by Piezo1, primarily Na^+^ and Ca^2+^, leads to membrane depolarization, effectively transforming mechanical stimuli into electrochemical responses^36^, ^37^. Notably, SERCA has been identified as an interacting protein with Piezo1, which inhibits channel activity by binding to a 14-residue intracellular linker situated between the pore and mechanotransduction modules, thereby playing a regulatory role in the adaptation to mechanical stress^170^. Additionally, Piezo1 is sensitive to bilayer tension in bleb membranes, which can be influenced by the cytoskeletal proteins and ECM stiffness^22^.

Murine studies have demonstrated that cardiac-specific knockout of Piezo1 impairs systolic function of the heart, whereas targeted overexpression of Piezo1 in cardiac tissue leads to severe heart failure and arrhythmias. These findings underscore the crucial role of Piezo1 homeostasis within myocytes in maintaining optimal cardiac performance^171^. *In vitro* experiments have shown that cyclic stretching (10% at 1 Hz) activates Piezo1, which in turn upregulates Nppb mRNA and protein expression, thereby promoting myofibroblast differentiation. Silencing Piezo1 reduces the stretch-induced expression levels of Nppb and TGF-β1 in cardiac fibroblasts, indicating that Piezo1 acts as a critical mechanosensitive ion channel mediating the activation of these cells in response to mechanical stretch^172^. Additionally, Piezo1 expression is elevated in cases of heart failure, and the binding of AngII to the AngII type 1 receptor (AT1) mediates the Erk1/2 pathway (Figure 1), enhancing the activity of the Piezo1 channel. This suggests that Piezo1 may play a pivotal role in ventricular remodeling during the progression of heart failure. Notably, treatment with the angiotensin receptor blocker (ARB) losartan can prevent the upregulation of Piezo1 in failing hearts^173^. Furthermore, another study employing a TAC induced pressure overload model in mice observed a significant increase in Piezo1 expression. Cardiac-specific Piezo1 knockout (Piezo1^Cko^) mice subjected to TAC exhibited markedly attenuated cardiac hypertrophy compared to controls. Further *in vitro* studies demonstrated that activation of Piezo1 promotes cardiomyocyte enlargement, an effect that can be suppressed by Piezo1 knockdown, the Yoda1 analog Dooku1, or the Piezo1 inhibitor GsMTx4 (Figure 1). Mechanistically, Piezo1 disrupts Ca^2+^ homeostasis by facilitating extracellular Ca^2+^ influx and inducing intracellular Ca^2+^ accumulation, thereby enhancing the activation of Ca^2+^-dependent signaling pathways, including calcineurin and calpain. This process is implicated in myocardial remodeling induced by mechanical stress^174^.

Pathological mechanical stress exerts a dual injurious effect on cardiac tissue by causing direct structural damage and indirectly activating inflammatory responses. The intertwined nature of inflammation and fibrosis contributes significantly to the progression of heart failure^70^. Braidotti *et al.* established that Piezo1 serves as a vital mechano-transducer, initiating and amplifying inflammatory cascades through Ca^2+^-dependent signaling pathways^166^. Activation of Piezo1 increases the permeability of the cell membrane to Ca^2+^, thereby elevating intracellular Ca^2+^ concentrations and activating Ca^2+^-sensitive kinases such as CaMK and PKC. These kinases, in turn, trigger downstream pro-inflammatory signaling pathways, including those mediated by IL-6, NF-κB, and MAPK. These pathways promote the expression and secretion of inflammatory mediators, exacerbating cardiac hypertrophy and inflammatory responses^166^, ^175^. The Piezo1-IL-6 axis plays a crucial role in amplifying these processes^175^. Yoda1, a selective agonist of Piezo1^176^, activates Piezo1 in cardiac fibroblasts and initiates Ca^2+^-dependent p38-MAPK signaling, leading to increased transcription and translation of the hypertrophic and profibrotic cytokine IL-6 (Figure 1). This suggests that mechanosensitive Piezo1 channels are functionally linked to paracrine IL-6 secretion, a mechanism that may be pivotal in regulating cardiac remodeling^177^. Sun *et al.* demonstrated that pathological mechanical stress in post-infarction hearts activates Piezo1-mediated signaling, increasing cardiac IL-6 receptor expression and phosphorylated STAT3 levels. This activation further propagates the neurogenic IL-6 inflammatory cascade, accelerating ventricular remodeling and the progression of heart failure. Notably, therapeutic strategies that target either Piezo1 or IL-6 signaling pathways have proved effective in attenuating these pathological processes^175^. Inflammatory responses can be triggered by the mechanical stresses inherent in heart failure^178^, and modulating mechanotransduction to suppress inflammation may offer a novel and potentially effective therapeutic approach for alleviating ventricular overload^179^.

Under conditions of abnormal mechanical stress, activation of the Piezo1 channel disrupts intracellular Ca^2+^ cycling dynamics, resulting in Ca^2+^ overload, activation of Ca^2+^/CaMKII, and subsequent aberrant Ca^2+^ release from the sarcoplasmic reticulum, which can induce arrhythmias^128^. CaMKII also exacerbates cellular injury by increasing mitochondrial Ca^2+^ uptake, ultimately leading to cardiomyocyte death^180^. Thus, mitochondrial responses to extracellular mechanical stress are at least partially mediated by mechanosensitive ion channels. In models of diabetic cardiomyopathy, mouse hearts with increased Ca^2+^ entry exhibited elevated calpain activity, enhanced phosphorylation of ERK1/2 and Drp1 (Figure 1), induced excessive mitochondrial fission, significantly reduced mitochondrial fusion, and increased ROS production, all contributing to mitochondrial dysfunction, cardiac fibrosis, and impaired cardiac function. However, cardiac-specific Piezo1 knockout improved cardiac contractile function and reduced fibrosis by normalizing calpain activity and restoring mitochondrial dynamics^181^. Additionally, the mechanically activated Piezo1 channel converts the mechanical stretching of cardiomyocytes into Ca^2+^ and ROS signaling. Studies indicate that both cardiac-specific ablation and overexpression of Piezo1 in murine models disrupt canonical Ca^2+^ and ROS signaling pathways, thus contributing to the development of cardiac dysfunction^171^, underscoring the importance of maintaining Piezo1 expression homeostasis for cardiac function.

### 4.3 TRP channels in mechanotransduction

The TRP channel family constitutes an essential group of mechanosensitive, Ca^2+^-permeable, nonselective cation channels that play critical roles in various cardiac functions including Ca^2+^ homeostasis, contractility, action potential modulation, mitochondrial operations, and adaptive remodeling^38^. Under pathological conditions including mechanical stress, hypoxia, or oxidative stress, TRP channels facilitate the transduction of extracellular stimuli into intracellular Ca^2+^ signals or biochemical cascades, which promote maladaptive responses in cardiomyocytes^182^. Current research indicates that most TRP channels do not directly respond to membrane tension but instead serve as secondary amplifiers within cellular mechanosensory signaling pathways. Nikolaev *et al.* have shown that members of the TRP family display limited responsiveness to direct membrane stretching, generally requiring cytoplasmic anchors or secondary messengers for mechanical activation^183^. This indirect mechano-sensitivity establishes TRP channels as pivotal integrators of signals, enhancing cellular mechanotransduction cascades during the progression of disease. Specifically, transient receptor potential vanilloid subtype 1 (TRPV1), transient receptor potential vanilloid 4 (TRPV4), transient receptor potential canonical 3 (TRPC3), and transient receptor potential canonical 6 (TRPC6) have been implicated in myocardial remodeling under mechanical stress, thereby constituting the primary focus of this section^165^, ^184^.

#### 4.3.1 TRPV1

TRPV1 channels, originally identified as capsaicin receptors, are nonselective cation channels that are permeable to Ca^2+^ and are highly expressed in sensory neurons and cardiac cells, including fibroblasts and cardiomyocytes^185^. The involvement of TRPV1 in pathological cardiac hypertrophy has attracted considerable attention. In models of myocardial hypertrophy induced by pressure overload, TRPV1 expression is significantly upregulated. Studies *in vivo* have demonstrated that knockout mice lacking the TRPV1 gene (TRPV1-/-) exhibit notable enhancements in cardiac function accompanied by reductions in hypertrophy, fibrosis, and apoptosis following TAC, substantiating the role of TRPV1 in the maladaptive remodeling induced by mechanical stress^186^. Although current research predominantly focuses on TRPV1's role in pressure overload scenarios, mechanical stress, a major factor in such overload, suggests a potential involvement of TRPV1 in mechanical stress-induced cardiac hypertrophy as well. Nevertheless, it is important to acknowledge that TRPV1 may exert dual regulatory effects. Lang *et al.* demonstrated that activation of TRPV1 by dietary capsaicin could mitigate the adverse effects of a high-salt diet on oxidative phosphorylation efficiency at Complex I (OXPHOS)^187^. Moreover, overexpression of TRPV1 in mice can protect against isoprenaline-induced proliferation of cardiac fibroblasts and collagen deposition, with these effects being mediated by Ca^2+^ influx and subsequent activation of eNOS activity^185^.

#### 4.3.2 TRPV4

TRPV4, an osmosensitive and mechanosensitive cation channel, is activated by arachidonic acid metabolites, primarily 5',6'-epoxyeicosatrienoic acid, and phorbol ester compounds^8^. This channel functions synergistically with Piezo1 to mediate Ca^2+^ influx in response to mechanical stimuli, suggesting that TRPV4 may operate downstream of the Piezo1 signaling pathways. Upon sensing mechanical stress, TRPV4 activates RhoA and the downstream effector ROCK, stabilizes actin filamin, and promotes the nuclear translocation of myocardin-related transcription factors (MRTFs) and serum response factor (SRF) (Figure 1). This activation of SRF-dependent transcription drives the expression of α-SMA and initiates the myofibroblast differentiation program^188^. Genetic ablation of TRPV4 reduces ECM rigidity, diminishes hypotonicity-induced Ca^2+^ influx, and inhibits myofibroblast transformation, even under conditions of TGF-β1 stimulation^189^. These findings underscore TRPV4 as a crucial mediator of both mechanical and biochemical signals during cardiac fibroblast activation^189^. In a diabetic animal model, TRPV4 expression is significantly upregulated, which in turn activates the TGF-β1/SMAD3 signaling pathway and promotes diabetic cardiac fibrosis. *In vitro* studies have shown that the TRPV4 antagonist HC067047 suppresses cardiac fibroblast proliferation, collagen I synthesis, and SMAD3 phosphorylation^190^. Collectively, these results indicate that TRPV4 contributes to pathological myocardial remodeling under conditions of mechanical stress.

#### 4.3.3 TRPC3

TRPC3, which is highly enriched in cardiac tissue, critically affects the pathogenesis of pressure overload-induced cardiac hypertrophy and heart failure^182^. Mechanical stretch induces TRPC3-mediated Ca^2+^ influx, leading to the activation of the nuclear factor of activated T-cells (NFAT) signaling pathway, which subsequently promotes the transcriptional upregulation of hypertrophic genes in cardiomyocytes^184^. Numaga-Tomita *et al.* have demonstrated that the microtubule-associated Rho guanine nucleotide exchange factor (GEF-H1) mediates TRPC3-dependent RhoA activation during mechanical stress, facilitating TGF-β-induced cardiac fibroblast-to-myofibroblast transition^191^ (Figure 1). These findings identify the TRPC3-GEF-H1 axis as a central mediator of fibrotic responses in both cardiomyocytes and fibroblasts under mechanical stress.

Beyond its canonical ion channel function, TRPC3 also regulates cardiac redox signaling through a channel-independent role as a positive regulator of reactive oxygen species (PRROS). Kitajima *et al.* demonstrated that TRPC3 forms a stable protein complex with Nox2, which stabilizes Nox2's enzymatic activity and expression. This TRPC3-Nox2 complex acts as a mechanosensitive redox signaling hub, amplifying ROS production in cardiomyocytes under mechanical stress conditions. The resulting oxidative stress activates profibrotic signaling cascades in both cardiomyocytes and cardiac fibroblasts, contributing to pathological remodeling. Notably, this PRROS mechanism of TRPC3 may represent a novel therapeutic target for HFpEF^192^.

#### 4.3.4 TRPC6

The primary physiological role of TRPC6 is the regulation of cation homeostasis, specifically sodium (Na^+^), potassium (K^+^), and Ca^2+^. Dysfunction of TRPC6 is closely linked to cardiovascular diseases^193^. In cardiomyocytes, Ang II activates phospholipase C (PLC), which produces diacylglycerol (DAG). DAG, in turn, directly inhibits TRPC6 channel activity. This inhibition leads to an influx of Ca^2+^, which, in conjunction with L-type Ca^2+^ channel activity, drives the activation of nuclear factor of activated T cells (NFAT)-dependent transcriptional programs that promote cardiac hypertrophy^194^. Experimental studies have shown that overexpression of TRPC6 exacerbates pathological remodeling by hyperactivating the calcineurin-NFAT pathways, thereby increasing the susceptibility to heart failure^195^. Notably, in models of HFpEF under hyperglycemic conditions, upregulation of TRPC6 facilitates the formation of a TRPC3-NOX2 complex, enhancing the production of ROS and accelerating disease progression^196^.

### 4.4 YAP/TAZ in mechanotransduction

The transcriptional coactivators YAP and PDZ-binding motif (TAZ) are central mediators in the Hippo signaling pathway. They play crucial roles in regulating gene expression critical for cell proliferation, differentiation, and apoptosis^135^. Typically, YAP forms a complex with TAZ to interact with members of the TEAD family of transcriptional enhancers (TEAD1-4), influencing downstream gene expression^22^. In its dephosphorylated state, YAP/TAZ translocates into the nucleus, where it binds to specific transcription factors and initiates the expression of downstream target genes^197^.

In the heart, YAP/TAZ function as central regulators of mechanical stress-induced cardiac hypertrophy and fibrosis. Under pathological loading conditions, activated YAP1 interacts with transcription factors such as TEAD1 to form a complex that downregulates the expression of mitochondrial dynamics regulators, including Drp1 and Mfn1 (Figure 2). This dysregulation impairs mitochondrial biogenesis, promotes pathological hypertrophy, and is associated with increased ROS production, DNA damage accumulation, and reduced cardiomyocyte proliferation. Pharmacological inhibition of YAP1 using verteporfin attenuates mitochondrial dysfunction and pathological hypertrophy in a murine model of chronic pressure overload^115^ (Figure 2). However, another study demonstrated that administration of verteporfin in a mouse model of ischemia-dependent cardiac fibrosis significantly reduced fibrosis and morphometric remodeling, albeit without improving cardiac function^198^. Consequently, the therapeutic potential of modulating YAP/TAZ for cardiac function in various clinical contexts warrants further investigation.

In cardiomyocytes under high glucose stress, YAP1 decreases ser127 phosphorylation and promotes extensive O-GlcNAcylation-mediated activation, which enhances forkhead box M1 (FOXM1) expression through the activation of AKT phosphorylation and subsequent inhibition of GSK3β. This cascade of molecular events leads to cardiomyocyte hypertrophy and fibrosis^199^. In the context of diabetic cardiomyopathy, YAP continuously activates TEAD1-dependent transcriptional programs, significantly exacerbating pressure overload-induced cardiac dysfunction. The inhibition of YAP substantially reduces leukocyte, macrophage, and neutrophil infiltration following pressure overload, suggesting that YAP contributes to heart failure by promoting cardiomyocyte dedifferentiation and inflammatory responses in diabetic hearts under stress^200^. Notably, prolonged activation of YAP has been associated with the progression of pressure overload-induced heart failure in HFD-fed murine models. These models simulate clinical conditions of obesity, metabolic syndrome, and insulin resistance observed in patients with HFpEF. Both pharmacological and genetic interventions targeting the YAP-TEAD pathway mitigate heart failure exacerbation induced by pressure overload in these models, indicating that YAP and TEAD are promising therapeutic targets for preventing cardiac dysfunction in HFpEF patients experiencing metabolic and concomitant mechanical stress overload^200^.

## 5. Summary and Outlook

HFpEF represents a heterogeneous clinical syndrome characterized by limited effective treatment options due to its complex pathophysiology. The heart functions as a mechanical pump, subjecting the cardiovascular system to intricate mechanical stresses with each contraction. While physiological mechanical stress is essential for normal cardiovascular function, aberrant mechanical stress contributes to the pathogenesis of cardiovascular diseases. In HFpEF, both macroscopic structural alterations and underlying molecular mechanisms are potentially associated with mechanical stress. This review explores the possible roles of mechanical stress in HFpEF pathology, including myocardial hypertrophy, fibrosis, systemic inflammation, endothelial dysfunction, metabolic dysregulation, and Ca^2+^ homeostasis impairment. We further elucidate the influence of mechanotransduction pathways on cardiac remodeling and the progression of heart failure while identifying potential therapeutic targets.

Although some mechanisms discussed may also apply to HFrEF, mechanical stress overload predominantly drives HFpEF pathogenesis, warranting focused investigation. Currently, sodium-glucose cotransporter protein 2 inhibitors (SGLT2i) are among the few effective therapeutic options for HFpEF. Preclinical evidence suggests that dapagliflozin can treat diabetic cardiomyopathy by inhibiting the expression of mechanical signal proteins under varying ECM stiffness. Emerging mechano-targeted therapies, including Piezo1 inhibitor GsMTx4, YAP1 inhibitor verteporfin, and TRPV4 antagonist HC067047, show promise in attenuating pathological cardiac remodeling. These agents have demonstrated favorable therapeutic potential in metabolic-associated heart failure models. Consequently, targeting mechanotransduction pathways may yield novel therapeutic strategies for HFpEF. It is important to note that substantial heterogeneity exists among HFpEF patients regarding etiology, comorbidities, and pathophysiological characteristics, which results in differential treatment responses. Therefore, the treatment of HFpEF should consider phenotype-specific personalized approaches to achieve optimal therapeutic outcomes.

## Abbreviations

ANP: Atrial natriuretic peptide
ARB: Angiotensin receptor blocker
ATP: Adenosine triphosphate
ACSL1: Acyl CoA synthetase-1
AT1: AngII type 1 receptor
Ang II: Angiotensin II
α-SMA: α-smooth muscle actin
AKT: Protein kinase B
BNP: Brain natriuretic peptide
BCAA: Branched-chain amino acid
BCAT2: Branched-chain amino acid transaminase 2
BCKD: Branched-chain α-ketoacid dehydrogenase
BCKAs: Branched-chain α-keto acids
Ca^2+^: Calcium ions
CaMK: Calmodulin dependent protein kinase
CoA: Acyl-coenzyme A
cyto c: Cytochrome c
Col1a: Collagen type I alpha 1
Col3a: Collagen type III alpha 1
CT-1: Cardiotrophin-1
CRP: C-reactive protein
cGMP: Cyclic guanosine monophosphate
CTGF: Connective tissue growth factor
Drp1: Dynamin-related protein 1
DAG: Diacylglycerol
ECM: Extracellular matrix
EndoMT: Endothelial-to-mesenchymal transition
eNOS: Endothelial nitric oxide synthase
ERK: Extracellular regulated protein kinases
ERK1/2: Extracellular signal-regulated kinase 1/2
FAK: Focal adhesion kinase
FOXM1: Forkhead box M1
GAPDH: Glyceraldehyde-3-phosphate dehydrogenase
gp130: Glycoprotein 130
HFpEF: Heart failure with preserved ejection fraction
HFmrEF: Heart failure with mid-range ejection fraction
HFrEF: Heart failure with reduced ejection fraction
HFSD: High-fat/high-sugar diet
IL-1β: Interleukin-1β
IL-13: Interleukin-13
IL-6: Interleukin-6
IL-18: Interleukin-18
ILK: Integrin-linked kinase
ITGBL1: Integrin beta-like 1
JNK: c-Jun N-terminal kinase
JAK1: Janus kinase1
LVEF: Left ventricular ejection fraction
LVEDP: Left ventricular end-diastolic filling pressures
LSS: Laminar shear stress
LAP: Latency-associated peptide
LTBP-1: TGF-β binding protein 1
LOX: Lysyl oxidase
LOXL: LOX-like
LIF: Leukemia inhibitory factor
LCFA: long-chain fatty acids
mtROS: Mitochondrial ROS
Mfn: Mitochondrial fusion protein
mtDNA: Mitochondrial DNA
MMP-2: Matrix metalloproteinase-2
mTORC1: Mechanistic target of rapamycin complex 1
MAPK: Mitogen-activated protein kinase
MPO: Myeloperoxidase
MICs: Mechanosensitive ion channels
MRTFs: Myocardin-related transcription factors
NRCMs: Neonatal rat cardiomyocytes
NOD: Nucleotide-binding and oligomerization domain
NLRP3: NOD-like receptor thermal protein domain-associated protein 3
NF-κB: Nuclear factor-kappa B
NO: nitric oxide
NADPH: Nicotinamide adenine dinucleotide phosphate
NOX: NADPH oxidase
Nrf2: Nuclear factor erythroid 2-related factor 2
NRF-1: Nuclear respiratory factor
NRCFs: Neonatal rat fibroblasts
NME1: NME/NM23 nucleoside diphosphate kinase 1
NFAT: Nuclear factor of activated T cells
Nfatc1: Transcription factor T nuclear factor 1
OSS: Oscillatory shear stress
OXPHOS: Complex I oxidative phosphorylation
PKA: Protein kinase A
p38 MAPK: p38 mitogen-activated protein kinase
PI3K: Phosphoinositide 3-kinase
P2X7: P2X purinoceptor 7
PKG: protein kinase G
PCr: Phosphocreatine
PTP: Permeability transition pore
PGC-1α: Peroxisome proliferator-activated receptor-γ coactivator-1α
PRROS: Positive regulator of reactive oxygen species
PLC: Phospholipase C
PARP-1: Poly (ADP-ribose) polymerase 1
ROS: Reactive oxygen species
RAC1: Rho family small GTPase 1
RhoA/ROCK: Ras Homolog Family Member A/Rho-associated coiled-coil-containing protein kinase
SGK1: Glucocorticoid regulated kinase 1
STAT1: Signal transducers and activators of transcription 1
SERCA: Sarcoplasmic/endoplasmic reticulum Ca²⁺ ATPase
SRF: Serum response factor
siRNA: Small interfering RNA
TAC: Transverse aortic constriction
TGF-β: Transforming growth factor beta
TIMP-2: Tissue inhibitor of MMP-2
TNF-α: Tumor necrosis factor α
TAK1: Transforming Growth Factor-β-Activated Kinase 1
TG2: Transglutaminase 2
Tyk2: Tyrosine kinase 2
TEAD: TEA domain transcription factor
TFAM: Mitochondrial transcription factor A
TRP: Transient receptor potential
TRPV1: Transient receptor potential vanilloid subtype 1
TRPC3: Transient receptor potential canonical 3
VSMCs: Vascular smooth muscle cells
VCL: Vinculin
YAP1: Yes-associated protein 1
